# P255: Study on the prevalence of healthcare associated infections in hospitals of Niger

**DOI:** 10.1186/2047-2994-2-S1-P255

**Published:** 2013-06-20

**Authors:** G Bawa-Alla, H Djibo

**Affiliations:** 1 Focal quality of care , Department of Public Health, Niger; 2 Department of Public Health, Faculty of Health Sciences, University of Niamey, Niamey, Niger

## Introduction

This study on the prevalence of nosocomial infection in long ignored Niger took place in three (3) national hospitals (HNN HNL and MIG) and two (2) regional reference centres (Maradi and Diffa). These institutions were selected on the basis of use of services and technical platforms.

## Objectives

To evaluate the prevalence of nosocomial infections in hospitals in Niger.

## Methods

A point prevalence survey making an assessment on "any given day" in each of the participating services, whose main characteristics were: Five hospitals totaling 1506 beds were concerned; 1040 hospitalized patients of whom 524 were male and 516 female bed occupancy 69.05%, 68 samples with microbial susceptibility testing were performed.

## Results

### Clinical Results

a prevalence of 7.3% of infected patients (76 patients) and 7.78% of nosocomial infections identified a ratio of infections / infected of 1.05, 93.42% of nosocomial infections are acquired in institutions; specialties most affected are general surgery (26%), gynecology and obstetrics (20%), general medicine (9.2%) and gastrointestinal surgery and pediatrics (7.9%).

### Microbiological Results

68 samples coming from the gynecology obstetrics services, general surgery, general medicine and Pediatrics, 51 samples out of 69 have at least one nosocomial strain (positive), 86% of the pus, urine and 71.42% 100% positive catheters; 70 pathogens consisting of 10 identified species, the four most commonlz isolated were: Staphylococcus aureus (41.41%), Pseudomonas (15.71%), Klebsiella (11.42%) and Eschericha coli (10%) species are most involved.

## Conclusion

This study has allowed us to make an inventory of the quality of care in hospitals and to expose the problem of nosocomial infections long ignored Care in Niger.

## Competing interests

None declared

